# Mesenchymal stem cells induce dynamic immunomodulation of airway and systemic immune cells *in vivo* but do not improve survival for mice with H1N1 virus-induced acute lung injury

**DOI:** 10.3389/fbioe.2023.1203387

**Published:** 2023-06-08

**Authors:** Yuan Tan, Yan Wang, Luciana Souza-Moreira, Chi Wang, Aidan B. P. Murray, Mahmoud Salkhordeh, Maria Florian, Lauralyn McIntyre, Duncan J. Stewart, Shirley H. J. Mei

**Affiliations:** ^1^ Regenerative Medicine Program, Ottawa Hospital Research Institute, Ottawa, ON, Canada; ^2^ Faculty of Medicine, University of Ottawa, Ottawa, ON, Canada; ^3^ Clinical Epidemiology Program, Ottawa Hospital Research Institute, Ottawa, ON, Canada

**Keywords:** mesenchymal stem cells, influenza A, immunomodulation, acute lung injury, viral infection, H1N1, immune cell profiling

## Abstract

**Introduction:** Influenza A virus (IAV)-induced acute lung injury (ALI) is characterized by pronounced proinflammatory activation and respiratory lung dysfunction. In this study, we performed deep immune profiling on airway and circulating immune cells to examine the effect of immunomodulation and therapeutic outcomes of mesenchymal stem cells (MSCs) therapy in mice with IAV-induced ALI.

**Methods:** Animals were inoculated intranasally with H1N1 IAV, followed by intravenous administration of vehicle, or human clinical-grade, bone marrow-derived MSCs 24-h later, and monitored for six days to evaluate the survival. In another set of animals, bronchoalveolar lavage (BAL) fluid and whole blood were collected three days after infection for flow or mass cytometry (CyTOF) immune profiling analysis.

**Results:** Immune cell population and phenotypic shifts in blood were mapped by CyTOF. Increases were observed in granulocytes and myeloid-derived cells in blood from vehicle-treated animals. While MSC treatment accentuated changes in these populations, naïve B, antibody-secreting B cells, and T cells were decreased in MSC-treated animals at day 3. Compared to sham animals, IAV infection induced a significant 5.5-fold increase in BAL total cell counts, including CD4^+^ and CD8^+^ T cells, CD19^+^ B cells, CD11b + Ly6G + neutrophils, and CD11b + Ly6C + monocytes. MSC treatment significantly decreased BAL total cell counts in IAV-infected mice, specifically the number of infiltrating CD4^+^ T cells and CD11b + Ly6G + neutrophils. In contrast, there were increases in CD8^+^ T cells, B cells, and monocytes in the alveolar space in MSC-treated animals. Phenotypic immune cell profiling of blood and BAL revealed a significantly higher proportion of the monocyte population with the M2 phenotype (CD206) in MSC-treated animals; however, this failed to confer protective effects in the survival of infected mice or reduce viral titer in the lung. Further investigation revealed that MSCs were susceptible to IAV infection, leading to increased cell death and potentially affecting their efficacy.

**Conclusion:** These findings provided *in vivo* evidence that MSCs promote the selective recruitment of immune cells to the site of infection during IAV infection, with reductions in proinflammatory phenotypes. However, MSCs offered no survival benefit in IAV-infected animals, possibly due to MSCs’ H1N1 IAV susceptibility and subsequent cell death.

## 1 Introduction

Acute respiratory distress syndrome (ARDS) is a clinical syndrome associated with lung inflammation, increased pulmonary vascular permeability, and blood and tissue hypoxemia (ARDS, 2012; Matthay et al., 2019). To date, ARDS remains one of the leading causes of death in critically ill patients, with a mortality rate of up to 40% (Bellani et al., 2016).

Influenza A virus (IAV) is a respiratory virus that primarily infects epithelial cells lining the respiratory tract of humans. Severe IAV infection can result in hospitalization for pneumonia and ARDS (Flerlage et al., 2021) and is the most common cause of pneumonia-related death (Herold et al., 2015; Peteranderl et al., 2016). Entry of IAV into host cells is mediated by binding of the viral surface glycoprotein, hemagglutinin (HA), to sialic acid (SA) receptors on the cell membrane (Skehel and Wiley, 2003). The distribution of different types of sialic acid receptors on host cells determines the tissue tropism and host range of IAV. Upon cell infection, IAV triggers a pronounced activation of the lung’s proinflammatory cascade. Uncontrolled, excessive cytokine production and immune cell activation are known to be central to the pathophysiology of influenza-induced ARDS (Yang et al., 2007). Treatments for severe IAV-induced ARDS are limited to the use of high-dose neuraminidase (NA) inhibitors and aggressive supportive care (Ventetuolo and Muratore, 2014; Herold et al., 2015), and new therapies are needed to improve the outcomes of patients with severe IAV infections.

Mesenchymal stromal/stem cells (MSCs) are a type of adult stem cells that have the ability to differentiate into various cell types and possess broad immunomodulatory properties (Le Blanc and Ringdén, 2007). MSCs have been proposed as a promising therapeutic strategy in viral pneumonia because of their immunomodulation and pro-resolution properties (Mei et al., 2007; Mei et al., 2016; Khoury et al., 2020). In the context of IAV infection, some preclinical studies showed that systemic administration of MSCs in IAV infection reduced virus-induced mortality, proinflammatory cytokines, and chemokines in the absence of reducing lung virus titers (Chan et al., 2016; Li et al., 2016), while others found that MSCs failed to provide protection (Darwish et al., 2013; Gotts et al., 2014). Additionally, concerns were raised over the susceptibility of MSCs to IAV infection, resulting in impaired cell functionality. Although known to potently inhibit T-cell and other immune effector cell functions *in vitro*, the knowledge of how MSCs function *in vivo* in the context of IAV-induced pneumonia remains unclear. Therefore, this study aimed to understand how MSCs may impact host immune responses during severe IAV-induced acute lung injury, with the goal of using this knowledge to design better therapeutic strategies in the future.

In our previous study, we revealed that viral-mimic priming augmented the immunomodulatory capacity of MSCs in an *ex vivo* co-culture system with whole blood from severe COVID-19 patients (Souza-Moreira et al., 2022). Therefore, we hypothesized that MSCs maintain their ability to modulate host immune cells *in vivo*, including both innate and adaptive immune cell populations, in the context of active H1N1 IAV infection. Here, we undertook a comprehensive analysis of systemic (whole blood) and local (bronchoalveolar space) immune profiling using an IAV model, in which MSCs were administered after the onset of infection. We also examined the susceptibility of MSCs to H1N1 IAV infection and assessed cell viability of IAV-infected MSCs.

## 2 Materials and methods

### 2.1 Bone marrow aspirates and MSC culture

Bone marrow aspirates were obtained from a commercial vendor (Lonza) or at The Ottawa Hospital (with informed written consent and ethical approval granted by the Ottawa Health Science Network Research Ethics Board, REB ID: 20120929-01H). Human bone marrow-derived MSCs (n = 3 different donors; 2 females and 1 male) were cultured, characterized, and cryopreserved, as described previously (McIntyre et al., 2018; Tan et al., 2019). MSCs have been characterized to meet all the criteria (plastic adherence, differentiation potential, and cell surface antigen expression) proposed by the International Society for Cellular Therapy (ISCT) (Dominici et al., 2006) as previously described (McIntyre et al., 2018; Tan et al., 2019). MSCs were thawed and cultured in Nutristem complete media (Biological Industries) for 24–72 h prior to being lifted and plated for *in vitro* assays or *in vivo* studies. MSCs were cultured and maintained at 37°C in a humidified incubator containing 5% CO_2_. All experiments used cells between passages 3 to 5.

### 2.2 IAV infection animal model

All animal experiments were conducted under the protocol approved by the University of Ottawa Animal Care Committee (ACC, institutional animal protocol #3371). Eight to nine-week-old female C57BL/6N mice were purchased from Charles River Laboratories (Saint-Constant, QC, Canada). They were allowed to acclimate in the animal care facility for one week prior to the experiment date. Animals were housed according to Canadian Council on Animal Care (CCAC) guidelines. All animals were housed in groups of either 3 or 4 in conventional breeding cages within the animal care facility. A standard chow diet was provided within the hopper of the cage to avoid soiling, and water was provided *ad libitum*. All cage bedding was changed on a weekly cycle. Animals were housed in a 20°C–23°C temperature-controlled room with a 12-h light, 12-h dark cycle and 40%–60% humidity. Daily wellness checks were conducted by the laboratory personnel, as well as the veterinary technicians on site.

**FIGURE 1:**
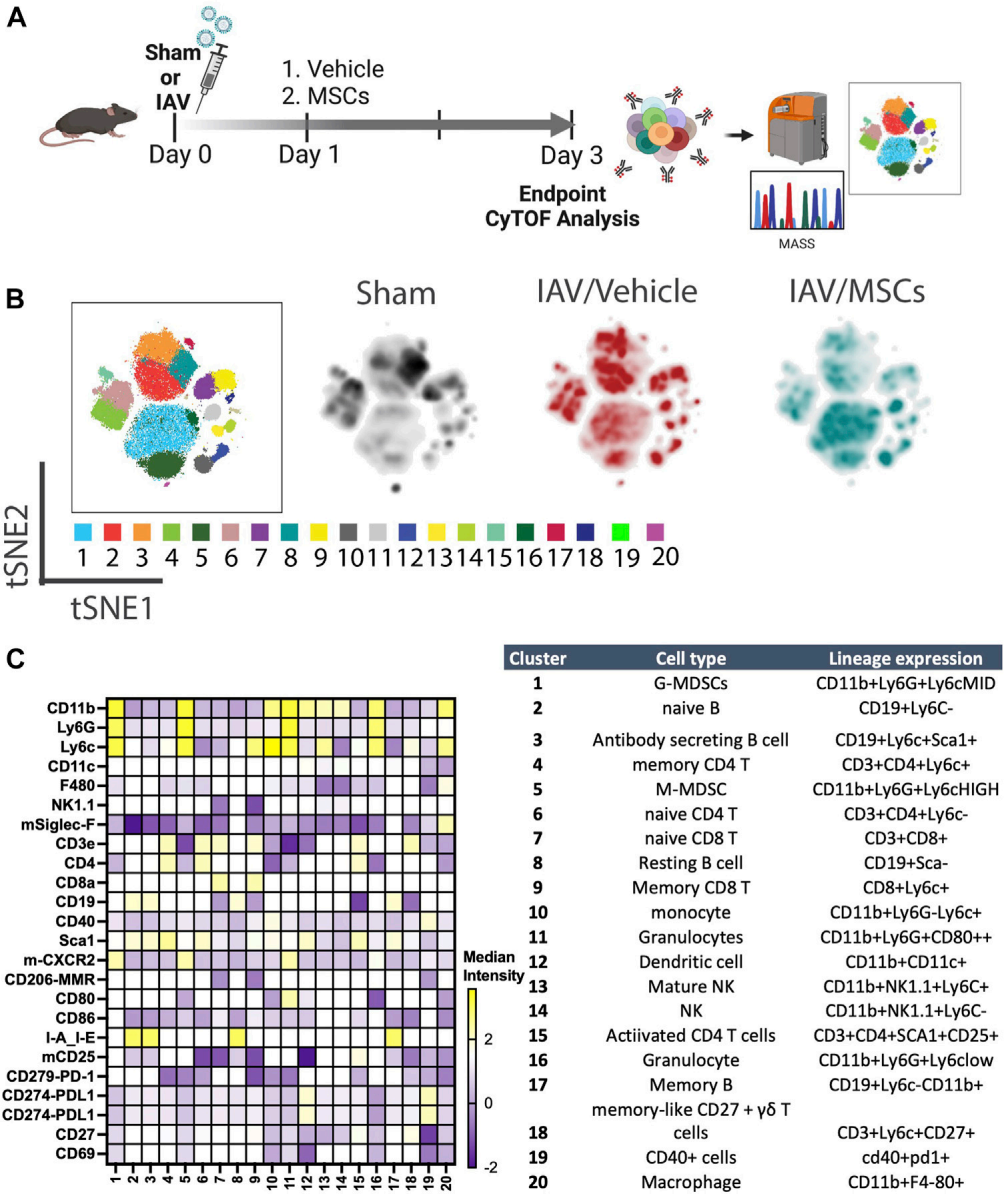
*In vivo* H1N1-induced ALI schematic and mass cytometry analysis of whole blood. **(A)** *In vivo* schematic of H1N1 IAV animal model with vehicle or MSCs treatment timeline, followed by endpoint tissue collection for the downstream CyTOF analysis. **(B)** Cell clusters were identified from CyTOF data by unsupervised clustering with the PhenoGraph algorithm. Each dot represents a cell (100,000 cells total). t-SNE plot overlaid with PhenoGraph clusters (the first map on the left), and density analysis was conducted (three map on the right). **(C)** Heatmap of 25-marker expression for different immune cell types with cluster identification (n = 6 of sham, n = 13 of IAV/vehicle-treated animals, and n = 11 of IAV/MSCs-treated animals).

For the experimental model, animals were anesthetized with isoflurane (1L/min O_2_ and 4% isoflurane) and then inoculated intranasally with 500 PFU (survival curve; 6 days) or 1000 PFU (3 days experiments) of IAV strain A/FM/1/47-MA (mouse-adapted H1N1 strain, a generous gift from Dr. Earl Brown at the University of Ottawa), diluted in 30 μL of sterile PBS. For hydration, mice received 40 mL/kg saline subcutaneously after IAV inoculation. Twenty-four hours after virus infection, animals were anesthetized with isoflurane (1L/min O_2_ and 1.5%–2% isoflurane), and a suspension of 2.5 × 10^5^ MSCs in Plasma-Lyte A (PLA) and 5% human albumin (100 µl total volume) or vehicle (PLA + 5% human albumin) was slowly infused via the jugular vein. After cell administration, the incision was closed with a sterilized surgical clip, and a topical pain-relieving cream was administered to the surgical site. Mice were monitored daily by trained laboratory personnel, as well as the veterinary technicians on site, until the end of the study. Note that animals were previously randomized (random.org; Randomness and Integrity Services Ltd.) for the treatment groups (vehicle or MSCs); cell infusion, animal wellness, and sample analyses were performed in a blinded fashion, with independent operators blinded for the group assignment. Any correlation between the treatment name and nature of the treatment was only revealed following the data collection and analysis period for graphing purposes. For mass cytometry immunoprofiling analysis, total numbers were n = 6 for sham, n = 13 for IAV/vehicle-treated animals, and n = 11 for IAV/MSCs-treated animals, see Figure 1A. For survival, a total of n = 15 for the IAV + vehicle group and n = 14 for the IAV + MSCs group were used (see Figure 6A).

### 2.3 Animal sample collection and processing

Mice were euthanized 3 days after IAV inoculation to evaluate the therapeutic efficacy by collecting samples for analysis. Mice were anesthetized with Ketamine (200 mg/kg) and Xylazine (10 mg/kg) cocktails through IP injection. Once the surgical plane was reached (mice not reactive to toe pinch), blood was collected from the inferior vena cava with a 50 mM EDTA-coated syringe.

For immune profile studies and cytokine analysis, bronchoalveolar lavage (BAL) was performed by inserting a catheter into the trachea and slowly injecting and aspirating 1 mL of saline three times. Total cell number was determined by 1:1 methylene blue counting by personnel blinded to the identity of animal injury/treatment. BAL was then centrifuged at 800 *g* for 10 min at 4°C, and supernatant (BAL fluid; BALF) was collected and stored at −80°C for further analysis. Cell pellets were resuspended in PBS and further fixed with paraformaldehyde 4% for flow cytometry analysis. Cytokine and chemokine levels in BALF were measured using a multiplex immunoassays kit (Bio-Rad) according to the manufacturer’s instruction and analyzed using Bio-Plex 200 System (Bio-Rad, United States). Total protein and IgM levels in BALF were measured using Bio-Rad DC (detergent compatible) Protein Assay (Bio-Rad) and a murine-specific IgM ELISA kit (Bethyl Laboratories), respectively, according to the manufacturer’s instruction. Note that BALF samples were incubated with 1% Triton X-100 at room temperature for one hour to deactivate IAV prior to the assay.

### 2.4 BAL cells staining

After fixation, cells were washed 3 times with staining buffer (PBS supplemented with 2% Fetal Bovine Serum). Cells were subsequently stained with anti-mouse CD11b, Ly6C, Ly6G, CD3, CD4, CD8, and CD19 (BD Biosciences) for 30 min at room temperature. Following incubation, cells were washed three times and resuspended in a staining buffer with CountBright Absolute Counting Beads (Thermo Fisher) for flow cytometry analysis (LSRFortessa cytometer, BD Biosciences). A detailed list of all antibodies is shown in Table 1.

### 2.5 Sample processing and antigen staining of mass cytometry-based immune cell profiling (CyTOF)

For CyTOF sample processing, 250 μL of mouse whole blood (EDTA) via caudal (inferior) vena cava was lysed in 350 μL of stable-lyse V2 and then fixed in 1 mL of stable-store V2 (Smart Tube Inc., San Carlos, US) as described in the user’s manual and stored at −80°C until further processing. Furthermore, whole blood samples were thawed and washed, followed by barcoding using Cell-ID 20-Plex Pd Barcoding Kit (Fluidigm). Up to four individual samples were barcoded for 30 min at room temperature. Cells were then washed and pooled for surface staining. Pooled cells were resuspended in diluted human Fc-block (BD bioscience) for 10 min and incubated with antibody staining cocktails for 30 min at room temperature. After incubation, cells were washed with staining buffer and fixed overnight in MaxPar Fix-I buffer with an iridium intercalator (Fluidigm). Immediately prior to data acquisition, samples were washed once with each of the cell staining buffer (Fluidigm) and cell acquisition solution (Fluidigm). Samples were then resuspended at a concentration of 5 × 10^5^ cells per mL in the cell acquisition solution containing EQ Four Element Calibration Beads (5:1 Ratio) (Fluidigm). The samples were acquired on a Helios Mass Cytometer equipped with a wide-bore sample injector at a rate of 300–500 events per second. After acquisition, repeat acquisitions of the same sample were concatenated as necessary and normalized using the Fluidigm software. Normalized FCS files were gated to exclude debris, doublets, and dead cells using Cytobank software.

All antibodies pre-conjugated to metal isotopes were obtained from Fluidigm Corporation (San Francisco, United States). All remaining antibodies were obtained from the indicated companies as purified antibodies, and in-house conjugation was done using the MaxPar X8 labeling kit (Fluidigm) following manufacturers’ recommended protocols. A detailed list of all antibodies used in this study is shown in Table 1.

**TABLE 1:** List of antibodies.

Antibody: Human	Supplier	Catalog #	RRID
RNase L (D4B4J) Rabbit mAb	Cell Signaling Technology	27281	AB_2798941
OAS1 (D1W3A)	Cell Signaling Technology	14498	AB_2798498
Rabbit mAb			
MX1 (D3W7I)	Cell Signaling Technology	37849	AB_2799122
Rabbit mAb			
IFITM3 (D8E8G) XP^®^ Rabbit mAb	Cell Signaling Technology	59212	AB_2799561
TRIM5α (D6Z8L)Rabbit mAb	Cell Signaling Technology	14326	AB_2798451
IFIT1 (D2X9Z)Rabbit mAb	Cell Signaling Technology	14769	AB_2783869
Monoclonal anti-β-actin, mouse, clone AC-15, ascites fluid	Sigma-Aldrich	A5441	AB_476744

Antibody: Mouse	Supplier	Catalog #	RRID
Anti-Mouse CD3e (145-2C11)-152Sm	Fluidigm	3152004C	N/A
Anti-Mouse CD4 (RM4-5)-145Nd	Fluidigm	3145002C	N/A
Anti-Mouse CD8a (53-6.7)-168Er	Fluidigm	3168003C	N/A
Anti-Mouse CD279 (RMP1-30)-159 Tb	Fluidigm	3159006C	N/A
Anti-Mouse CD11b (M1/70)-148Nd	Fluidigm	3148003C	N/A
Anti-Mouse CD11c (N418)-209Bi	Fluidigm	3209005C	N/A
Anti-Mouse I-A/IE (M5/114.15.2)-174Yb	Fluidigm	3174003C	N/A
Anti-Mouse/Rat CD40 (HM40-3)-161Dy	Fluidigm	3161020C	N/A
Anti-Mouse CD80 (16-10A1)-171Yb	Fluidigm	3171008C	N/A
Anti-Mouse CD86 (GL1)-172Yb	Fluidigm	3172016C	N/A
Anti-Mouse Ly-6C (HK1.4)-162D	Fluidigm	3162014C	N/A
Anti-Mouse Ly-6G (1A8)-141Pr	Fluidigm	3141008C	N/A
Anti-Mouse CD206/MMR (C068C2)-169 Tm	Fluidigm	3169021C	N/A
Anti-Mouse CD64 (X54-5/7.1)-151Eu	Fluidigm	3151012C	N/A
Anti-Human/Mouse CD27 (LG.3A10)-150Nd	Fluidigm	3150017C	N/A
Anti-Mouse Ly-6A/E (Sca-1) (D7)-164Dy	Fluidigm	3164005C	N/A
Anti-Mouse CD45 (30-F11)-89Y	Fluidigm	3089005C	N/A
Anti-Mouse CD274/PD-L1 (10F.9G2)-153Eu	Fluidigm	3153016C	N/A
Anti-Mouse F4/80 (BM8)-146Nd	Fluidigm	3146008C	N/A
Anti-Mouse NK1.1 (PK136)-170Er	Fluidigm	3170002C	N/A
Anti-Mouse CD19 (6D5)-166Er	Fluidigm	3166015C	N/A
Anti-Mouse CD69 (H1.2F3)-143Nd	Fluidigm	3143004C	N/A
Anti-Mouse CD45 (30-F11)-89Y	Fluidigm	3089005C	N/A

### 2.6 Mass cytometry data analyses

Files were processed following Fluidigm recommendation, including randomization and normalization using EQ Beads signal. Files were then concatenated, debarcoded, and randomized according to Fluidigm’s instructions using the CyTOF Software. Gating to identify and export single cells was completed in Cytobank (Kotecha et al., 2010). Clustering analysis was completed with either FlowSOM (Cytobank) (Van Gassen et al., 2015) or PhenoGraph (Levine et al., 2015) as stated as part of the R Bioconductor package Cytofkit (Chen et al., 2016) and Flowjo X using the markers listed in the Key Resource Table with up to 50,000 cells per sample. The transformation method used was cytofAsinh, and the visualization method was t-SNE. The t-SNE map overlaid with PhenoGraph-defined cell populations (Supplementary Figures S3B, S4B) was generated using the ggplot2 package in RStudio (open-source) and FlowJo (BD Biosciences).

### 2.7 Automated western blot analysis

MSCs were seeded into 24-well plates (100,000 cells/well) and treated the day after with 10% of BALF obtained from the control (sham) or IAV-infected (sick) animals; in parallel, cells were also infected with IAV (A/FM/1/47-MA) at a multiplicity of infection (MOI) of 0.1. After 24h, cell lysate was collected by adding a sample lysis buffer cocktail containing proteinase inhibitor cocktail (Sigma-Millipore) and cell scraper. Protein quantification was performed using the Bradford reagent (Bio-Rad). The target protein signal was measured by an automated capillary electrophoresis-based western blotting system (Jess, ProteinSimple, San Jose, CA, United States). In summary, samples were diluted to 1µg/µl total protein in simple western sample buffers from the 12–230 kDa Jess Separation Module cartridge kit (cat# SM-W004), heated at 95°C for 5 min, and then loaded to the plate. All Jess simple western system experiments were conducted according to the manufacturer’s instructions. Compass for SW software (ProteinSimple) was used to analyze and quantify protein band intensities. A detailed list of all antibodies used in this study is shown in Table 1.

### 2.8 Sialic acid detection on MSCs

150,000 MSCs were washed three times with staining buffer (PBS supplemented with 2% fetal bovine serum). Cells were stained with 100 μL of 100 μg/mL Maackia Amurensis Lectin (MAA) conjugated with Dylight 649 (EY Laboratories, DY649-7801-1) and Sambucus Nigra (Elderberry Bark) Lectin (SNA, EBL) conjugated with fluorescein (FITC) (Thermo Fisher, L32479) for 30 min at room temperature. Following incubation, cells were washed three times and resuspended in a staining buffer for flow cytometry analysis (Attune NxT Flow Cytometer, Invitrogen).

### 2.9 *In vitro* virus infection assays

MSCs were seeded into 48-well plates with a seeding density of 15,000 cells/per well. The day after, cells were infected by H1N1 IAV (A/FM/1/47-MA) at an MOI of 1 diluted in OptiMEM(100 μl/well). After 1 h, the infection media were removed, and cells were washed twice with PBS. MSCs were cultured in Nutristem complete media (Biological Industries) at 37°C for 24 h, 48 h, and 72 h. For LDH assay and testing virus titer, conditioned media of each group were collected at each time point, and then cells were washed with PBS and fixed using 4% paraformaldehyde (PFA). Lactate dehydrogenase (LDH) measurement was performed using the CytoTox 96^®^ Non-Radioactive Cytotoxicity Assay (Promega). For virus titer, IAV RNA was isolated from conditioned media using the QIAamp Viral RNA Mini RNA isolation kit (QIAGEN), and the qPCR was performed using QuantiTech Virus Kit (QIAGEN) and analyzed in CFX384 Thermocycler (Bio-Rad). Cell images were obtained using Incucyte S3, objective 20x (Sartorius). For nuclei counting of attached cells, DAPI (4′,6-diamidino-2-phenylindole, Thermo Fisher) staining was used to identify nuclei. The fluorescent images were captured and analyzed (Cellomics ArrayScan VTI HCS Reader, Thermo Fisher Scientific, CA, United States). For publication purposes, the brightness of all images was enhanced at the same percentage (+20%) to optimize visualization.

### 2.10 Statistical analysis

Statistical analysis was performed using GraphPad PrismV9.0 software (GraphPad Software). Numerical data are presented as mean ± SEM unless otherwise stated. Analysis was conducted using a two-tailed t-test. Multiple groups were analyzed by one-way ANOVA followed by Sidak’s or Dunnett’s multiple comparison test unless otherwise stated. For the analysis of cytokine data, logarithmic transformation was performed to normalize the data distribution before conducting ANOVA. Statistical significance was set at *p* < 0.05.

## 3 Results

### 3.1 MSCs induced immunomodulatory changes in circulating innate immune cell population in an IAV-induced acute lung injury (ALI)

**FIGURE 2:**
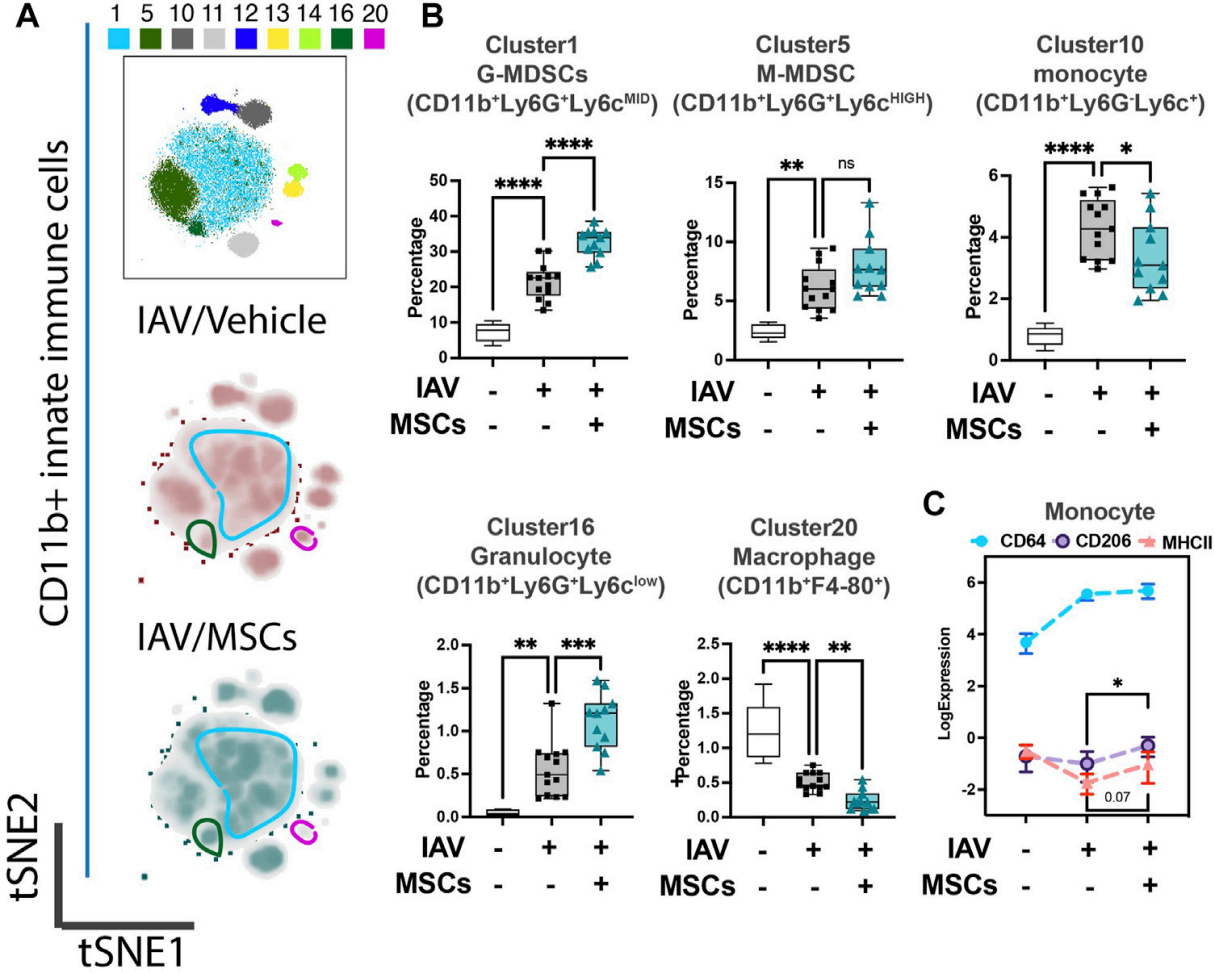
MSCs reshape innate host immune responses in circulation (whole blood) of IAV-infected mice. **(A)** Subsequent tSNE analysis of CD11b+ innate immune cell population, overlaid with PhenoGraph clustering identified in Figure 1A. **(B)** Quantification of cell subpopulations, identified by unsupervised clustering with PhenoGraph algorithm of CyTOF data, in whole blood isolated from IAV-infected mice (Supplementary Figure S1C). Analysis showed modulatory effects of MSCs on host innate immune responses during IAV infection. **(C)** Quantification of CD64, CD206, and MHCII expression from CyTOF data showed increased expression of CD206 in MSC-treated groups. n = 6 of sham, n = 13 of IAV/vehicle-treated, and n = 11 of IAV/MSCs-treated animals, with the data shown as box-and-whisker plots **(B)**, or symbol = mean ± SEM **(C)**. Group comparisons were analyzed by one-way ANOVA with Dunnett’s *post hoc* test. ∗*p* < 0.05, ∗∗*p* < 0.01, ∗∗∗*p* < 0.005, and ∗∗∗∗*p* < 0.001. See also Supplementary Figure S1.

**FIGURE 3:**
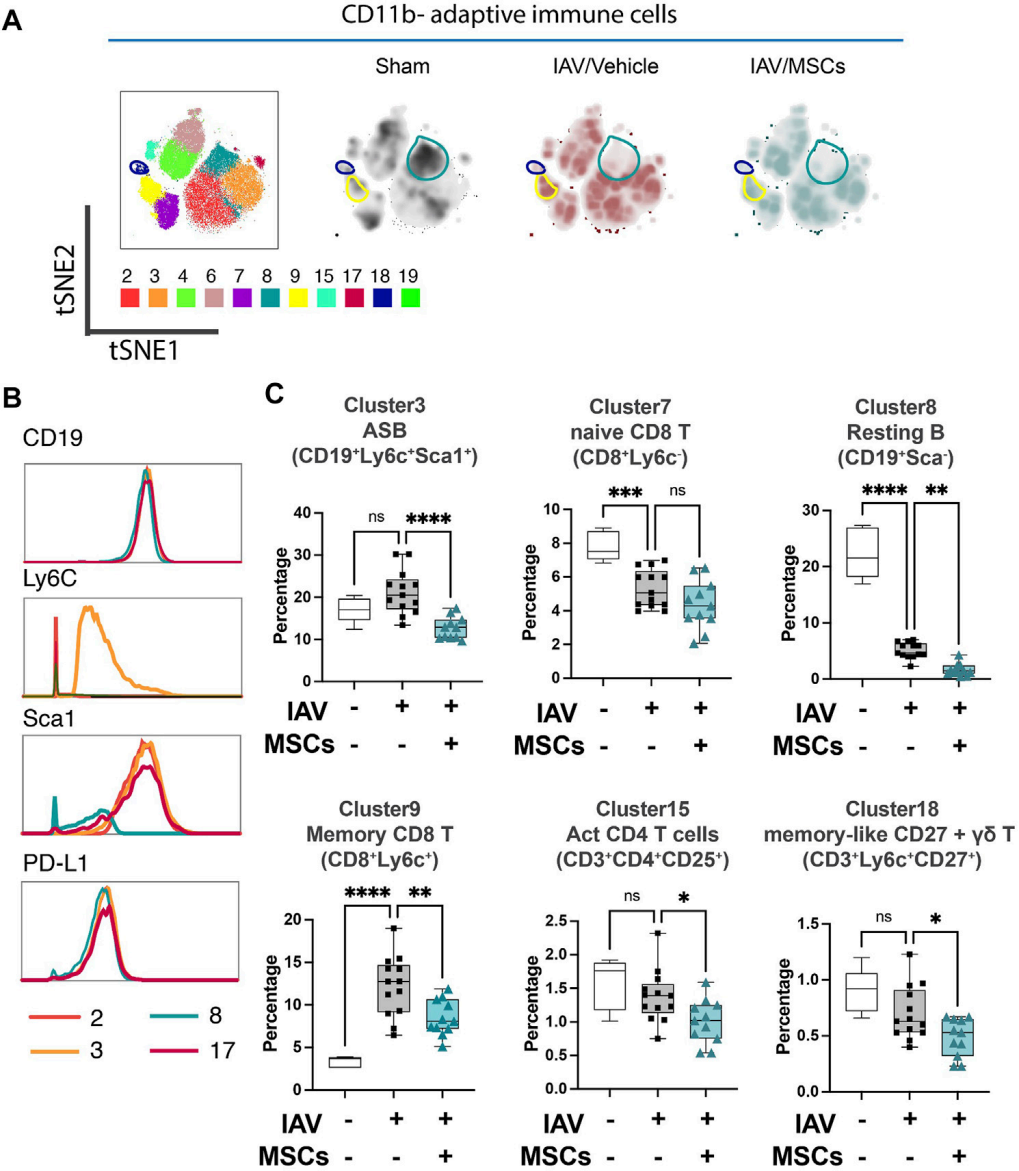
MSCs alter adaptive immune responses in circulation (whole blood) in IAV-infected mice. **(A)** tSNE analysis of CD11b-adaptative immune cell population, overlaid with PhenoGraph clustering identified in Figure 1A. **(B)** Histogram of various B-cell-related surface marker expression of clusters 2, 3, 8, and 17. **(C)** Quantification of cell subpopulations, identified by unsupervised clustering with PhenoGraph algorithm of CyTOF data, in whole blood isolated from IAV-infected mice. n = 6 of sham, n = 13 of IAV/vehicle-treated animals, and n = 11 of IAV/MSCs-treated animals, with the data shown as box-and-whisker plots. Group comparisons were analyzed by one-way ANOVA with Dunnett’s *post hoc* test. ∗*p* < 0.05, ∗∗*p* < 0.01, ∗∗∗*p* < 0.005, and ∗∗∗∗*p* < 0.001. See also Supplementary Figure S2.

We first investigated whether MSCs can improve host immune responses when exposed to a virus-infected host microenvironment *in vivo*. Mice were challenged with IAVs, followed by treatment with vehicles or MSCs (Figure 1A). Immune cells in whole blood were profiled at day 3 after infection by CyTOF, followed by t-SNE analysis and PhenoGraph clustering, revealing 20 distinct populations (Figures 1B, C). The density analysis of the t-SNE maps demonstrates dynamic shifts of circulating immune cell population in IAV-infected mice (vs. sham, Figure 1B), while the expression level of individual cluster’s surface markers is represented in a heatmap (Figure 1C). To analyze the modulation of both innate and adaptive immune cell populations, CD11b expression was used to further subgroup the CD11b+ innate cells (Figure 2) and CD11b-adaptive immune cells (Figure 3) in the t-SNE analysis. In Figure 2Aand Supplementary Figure S1A, the innate immune cell population was clustered into t-SNE maps, overlaid with clusters 1, 5, 10, 11, 12, 13, 14, 16, and 20. In vehicle-treated IAV animals, there was significant enrichment of granulocytic (cluster 1) and monocytic (cluster 5) myeloid-derived cells, monocytes (cluster 10), granulocytes (cluster 16), and dendritic cells (DCs, cluster 12) compared to that in the sham animals (Figure 2B; Supplementary Figure S1B). On the contrary, circulating macrophage (cluster 20) was significantly depleted (*p* < 0.001 vs. sham, Figure 2B). With MSC administration, we observed distinctive changes in innate cell populations compared to vehicle-treated animals (shown in Figure 2B; Supplementary Figure S1B). MSCs administration significantly potentiated further increase of granulocytic (cluster1) myeloid-derived cells and granulocytes (cluster 16) and decreased circulating macrophage (cluster 20) while driving a reduction of monocyte (cluster 10) in circulation (Figure 2B). Natural killer (NK) (clusters 13 and 14) cells and increased DCs induced by IAV (cluster 12) have no significant changes with MSCs administration (Supplementary Figure S1B). When examining the phenotypic marker profile, the monocyte population in whole blood exhibited a greater M2 (CD206+) and less immune tolerance (high MHC-II) phenotype after MSCs (Figure 2C), compared to that in vehicle-treated ones.

### 3.2 MSCs modulated circulating adaptive immune cell population in IAV infection

When analyzing the adaptive immune cells (CD11b-), PhenoGraph clusters 2, 3, 4, 6, 7, 8, 9, 15, 17, 18, and 19 were analyzed into the tSNE map (Figure 3A). Density maps further revealed distinctive enrichment and depletion of B- and T-cell populations, as shown in Figure 3A, and different B-cell subpopulation were identified using markers including Ly6C and Sca1(Figure 3B). With H1N1 infection, a marked depletion of naïve T cells (CD8^+^ T cell in cluster 7) and naïve (resting) B cells (cluster 8) was observed, while MSCs further promoted these changes (Figure 3C). Moreover, while there was a marked increase of memory CD8^+^ T cells (cluster 9) seen in the whole blood of IAV animals (vs. sham), MSCs treatment decreased the levels of these circulating immune cell clusters (vs. IAV/vehicle-treated, Figure 3C; Supplementary Figure S2). Interestingly, percentages of antibody-secreting B cells (ASB) (cluster 3), activated CD4 T cells (CD25^+^, cluster 15), and gamma–delta T cells (cluster 18) were not significantly altered in vehicle-treated IAV animals (not significant vs. sham). These cell populations were significantly lowered by MSCs (vs. IAV/vehicle-treated, Figure 3C). Taken together, MSCs were able to induce early alterations in both innate and adaptive immune responses during H1N1 IAV infection.

### 3.3 Modulation of immune cell airspace infiltration by MSCs in the airway of IAV-infected lungs

**FIGURE 4:**
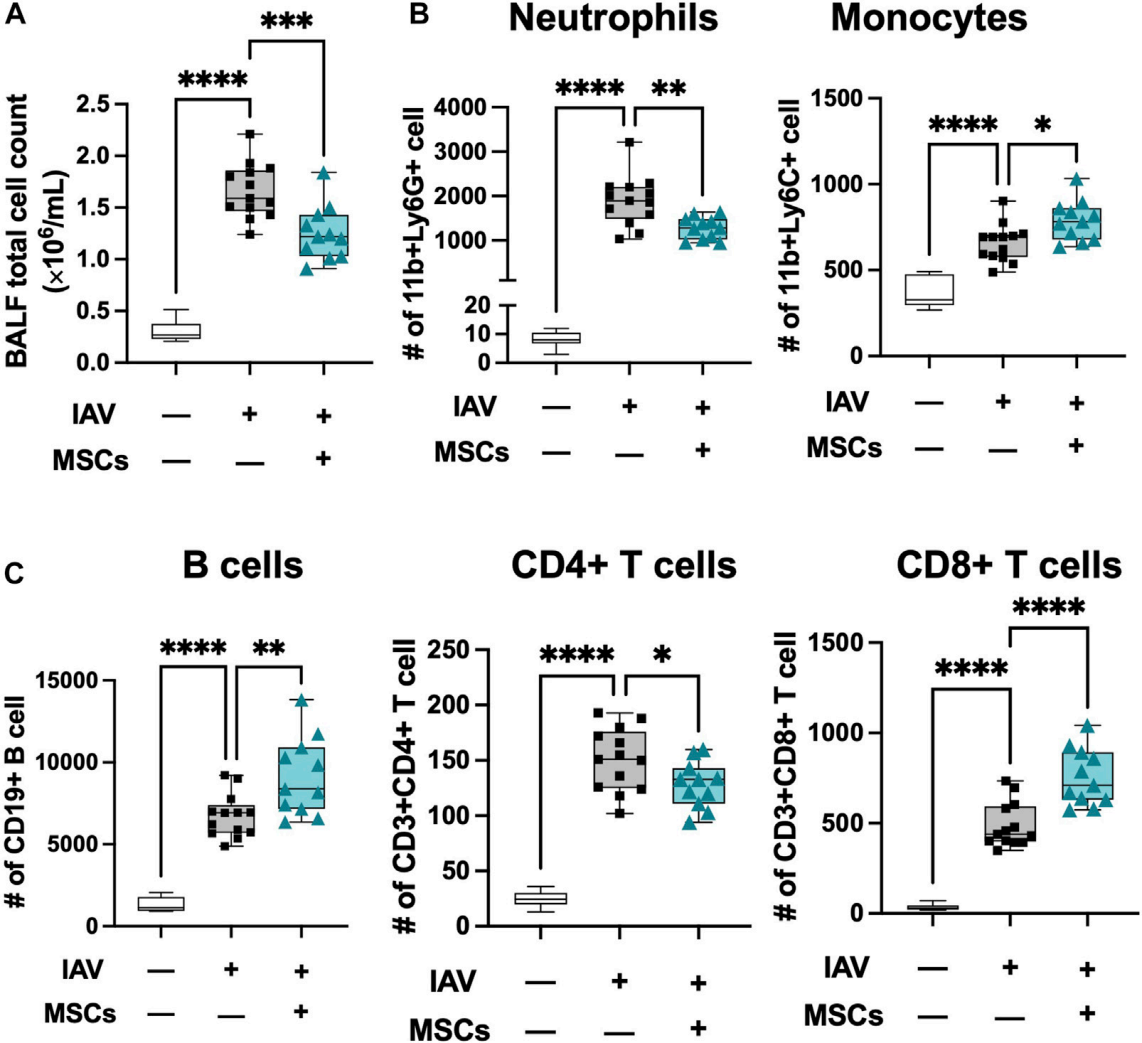
MSCs modulate immune cell infiltration into localized bronchoalveolar spaces. **(A)** Cell counts of total BAL infiltrating cells. Flow cytometry analysis of cells collected from BAL of IAV-infected mouse lung with or without MSCs treatment showed immune modulation on **(B)** innate immune cell (neutrophil and monocyte) and **(C)** adaptive immune cell (B cells, CD4^+^ T cells, and CD8^+^ T cells) by MSCs. n = 6 of sham, n = 13 of IAV/vehicle-treated animals, and n = 11 of IAV/MSCs-treated animals with the data shown as box-and-whisker plots. Group comparisons were analyzed by one-way ANOVA with Dunnett’s *post hoc* test. ∗*p* < 0.05, ∗∗*p* < 0.01, ∗∗∗*p* < 0.005, and ∗∗∗∗*p* < 0.001.

Next, we profiled changes in immune cell infiltration to bronchoalveolar space. Total cell counts in BAL were significantly elevated in IAV-infected animals (vs. sham), coinciding with early response to infection, and these were significantly decreased by MSCs treatment (vs. IAV/vehicle-treated, Figure 4A). The absolute number of neutrophils, monocytes, B cell, CD4^+^, and CD8^+^ T cells were all elevated in vehicle-treated mice (Figures 4B and C). MSCs significantly decreased the number of CD11b + Ly6G + neutrophils (Fig. 4B). In contrast, MSC-treated animals showed a significantly increased number of CD11b + Ly6C + monocytes (Figure 4B), CD8^+^ T, and CD19^+^ B cells (Figure 4C) in the BAL, corresponding to a depletion of these populations in circulation (Figures 2B, 3C), suggesting MSCs may accelerate their recruitment into the alveolar airspace.

### 3.4 Upregulated antiviral protein expression in MSCs upon exposure to IAV-infected BALF

**FIGURE 5:**
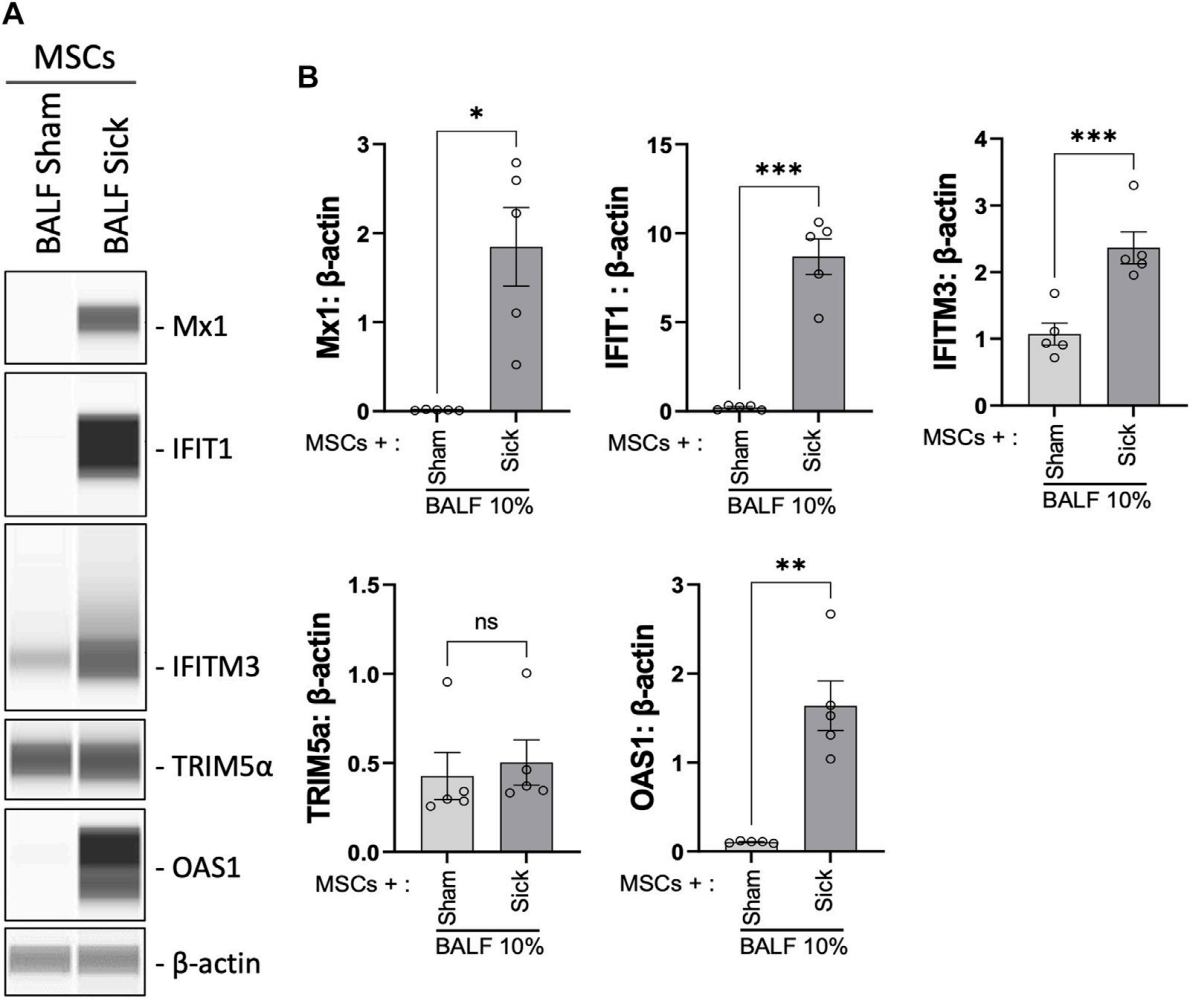
Upregulation of antiviral protein expression in MSCs after being exposed to bronchoalveolar lavage of H1N1 IAV-infected animal. **(A)** Automated western blot analysis showed increased antiviral protein expressions in MSCs after *in vitro* exposure to BALF (BALF, 10%) from sham or sick (IAV-infected) mice. **(B)** Densitometry analysis of protein expression detected using automated western blot. n = 5 with the data shown as mean ± SEM. ∗*p* < 0.05, ∗∗*p* < 0.01, and ***p < 0.005 using two-tailed t-test.

We previously showed that MSCs upregulated antiviral protein expression induced by exposure to the viral mimic, poly (I:C) (Souza-Moreira et al., 2022). Next, the expression level of a range of antiviral proteins was examined in MSCs after exposure to BALF collected from H1N1 IAV-infected animal *ex vivo*. Upon stimulation with H1N1 IAV-infected BALF (BALF sick), MSCs exhibited marked elevation of Mx1, IFIT1, IFITM3, and OAS1 expression, compared to that in control MSCs or cells treated with BALF from sham animals (BALF sham, Figures 5A, B).

### 3.5 MSCs did not improve survival of IAV-induced ALI

**FIGURE 6:**
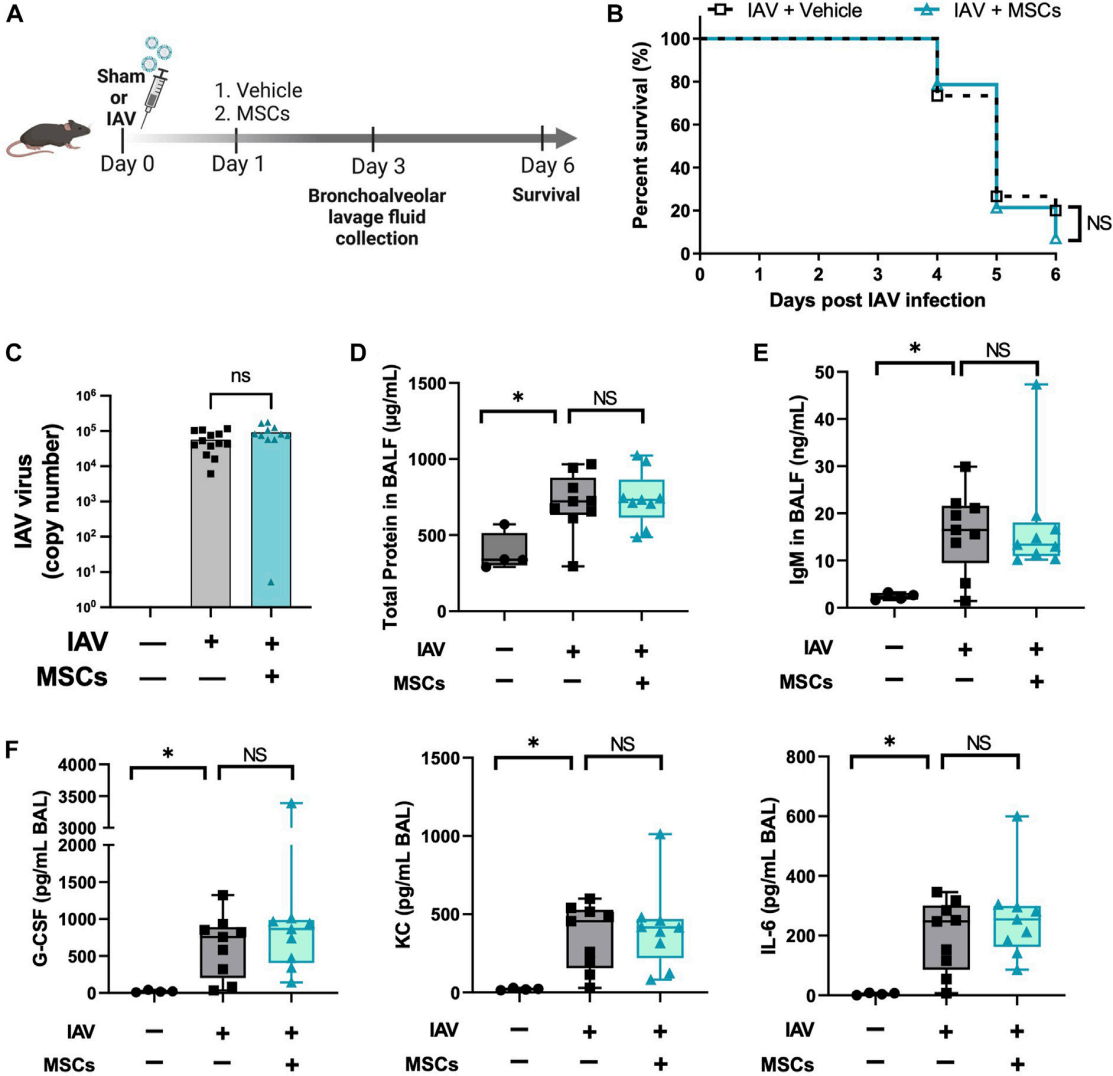
MSCs did not rescue IAV-infected mice. **(A)** *In vivo* schematic of the study. Mice received 500 PFU of influenza A intranasally. One day after virus infection, mice received MSCs or vehicle treatment via the jugular vein and were monitored for 6 days after virus inoculation. **(B)** Percentage of animal survival data in vehicle vs. MSCs-treated IAV mice over 6 days. **(C)** Virus titer in mice BALF at day 3 after infection. **(D)** Total protein levels in BALF at day 3 after infection. **(E)** IgM level in BALF at day 3 after infection. **(F)** Cytokines levels (G-CSF, KC, and IL-6) in BALF at day 3 after infection. n = 9–15 for IAV/vehicle group or IAV/MSCs group, with the data shown as mean ± SEM. Survival result was analyzed by log-rank test **(B)**. Group comparisons were analyzed by one-way ANOVA with Dunnett’s *post hoc* test **(D–F)**. ∗*p* < 0.05, ∗∗*p* < 0.01, ∗∗∗*p* < 0.005, and ∗∗∗∗*p* < 0.001.

With the evidence showing that MSCs maintain their capacities to modulate immune cells *in vivo* during H1N1 infection, we further tested the therapeutic potential of MSCs in protecting mice from H1N1-induced mortality (Figure 6A). Unexpectedly, no improvement in survival rate was observed from the MSC-treated group compared to vehicle-treated IAV mice (Figure 6B). Corresponding to the survival result, MSC- and vehicle-treated animals showed similar levels of virus load in BALF at day 3 after virus infection (Figure 6C). An aggravated pulmonary vascular permeability was observed in vehicle-treated IAV mice with a significant increase in total protein and IgM in BALF (Figures 6D, E). Additionally, BALF from vehicle-treated IAV mice exhibited a significant increase in cytokine levels (Figure 6F). However, MSCs did not induce changes either in the alveolar–capillary membrane barrier integrity (Figures 6D, E) or in the cytokine levels in BALF compared with vehicle treatment (Figure 6F). Taken together, although MSCs exhibited significant immunomodulatory activities in the immune system, our results showed that MSCs did not prevent mortality or lung injury induced by H1N1 IAV infection in mice.

### 3.6 MSCs highly expressed H1N1 binding receptor and underwent cell death upon IAV infection

**FIGURE 7:**
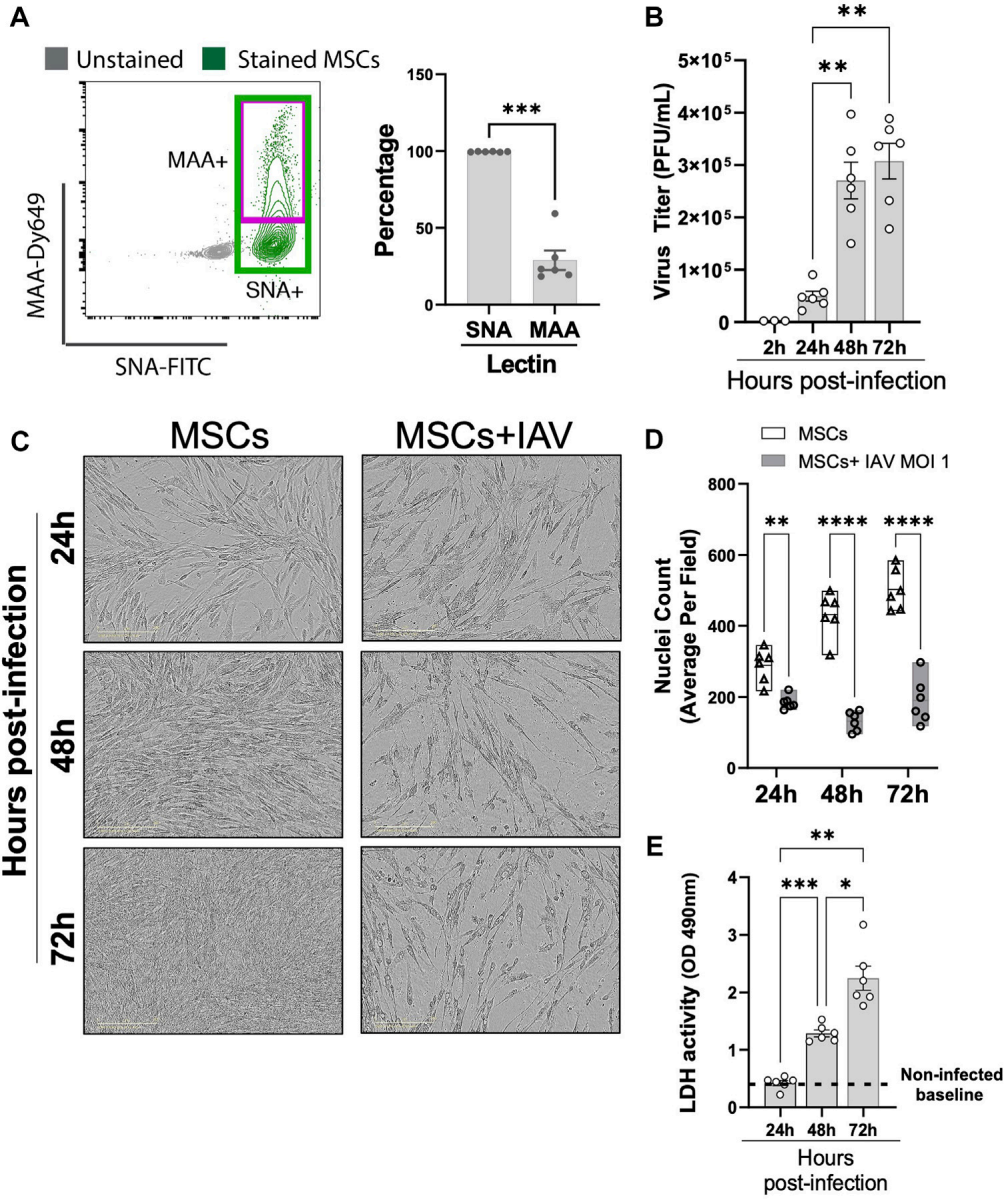
Viral replication in H1N1-infected MSCs. **(A)** MSCs were stained with fluorescein (FITC) conjugated Sambucus Nigra Lectin (SNA, EBL) and Dylight 649 conjugated Maackia Amurensis Lectin (MAA), followed by analyzing via flow cytometry for detecting the presence of salic acid on MSCs. n = 6 with the data shown as mean ± SEM, ∗∗∗*p* < 0.0005. **(B)** Viral titer measured by copy number (determined by qPCR) in the conditioned media harvested from MSCs infected with IAV. n = 3–6 with the data shown as mean ± SEM, ∗∗*p* < 0.005. **(C)** Representative phase microscopic images of MSCs with or without IAV H1N1 infection (MOI 1). **(D)** Nuclei counting of DAPI-stained cell images. Graph shows average per field; a total of 36 fields were imaged per well for each condition per individual experiment. n = 6 with the data shown as mean ± SEM; ∗∗*p* < 0.005 and ∗∗∗∗*p* < 0.0005. **(E)** LDH activity showing that MSCs underwent cell death after H1N1 infection (MOI 1) over time. n = 6 with the data shown as mean ± SEM, ∗*p* < 0.05, ∗∗*p* < 0.005, and ∗∗∗*p* < 0.0005. Group comparisons were analyzed by one-way ANOVA with Dunnett’s *post hoc* test (A, B, and E) and Sidak’s *post hoc* test (D).

Having established that MSCs do not protect against animal mortality *in vivo*, we next investigated whether IAVs can infect MSCs. Given that MSCs were previously tested in virus-induced ALI using different IAV strains (mammalian H1N1 and avian H5/H9) (Darwish et al., 2013; Chan et al., 2016) with different degrees of therapeutic efficacy, we examined whether there is a differential expression pattern of α-2,6-linked SA (receptor for mammalian H1N1, by SNA staining) and α-2,3-linked SA (receptor for avian H5 or H9 IAV strains, by MAA staining) on MSCs. Our data demonstrated that MSCs expressed high levels of H1N1 IAV receptor, α-2,6-linked SA (shown as SNA + stained cells), while a significantly smaller population of MSCs expressed level of avian IAV receptor (α-2,3-linked SA, shown as MAA + stained cells) (Figure 7A). Direct H1N1 IAV infection still led to upregulated antiviral protein (Mx1, IFIT1, IFIMIT3, TRIM5a, and OAS1) in MSCs (Supplementary Figure S3), corroborating with our data of increased antiviral protein expression by MSCs after being exposed to H1N1-infected animal BALF (Figure 5). Interestingly, we observed a significant increase in viral titer in conditioned media of MSCs at 48 h after infection (at 2.70 ± 0.35 × 10^5^ PFU/mL), compared to 24 h (at 4.96 ± 0.95 × 10^4^ PFU/mL, respectively, *p* < 0.01; Figure 7B). The high virus load plateaued at 72 h (3.07 ± 0.34 × 10^5^ PFU/mL), possibly due to the high cytopathic effect induced by the H1N1 IAV strain. Indeed, marked morphological changes (Figure 7C) and reduced adherent MSCs (identified by nuclei staining, Figure 7D) were observed via microscopy after 24-h IAV infection. Additionally, the LDH level, which is released by cells upon plasma membrane damage or cell death, was significantly elevated at 48 h after infection (Figure 7E). These results confirmed that the H1N1 strain used in our study can infect and kill MSC, which might limit therapeutic efficacy of MSCs treatment seen *in vivo*.

## 4 Discussion

In a model of H1N1 IAV-induced ALI, we showed that intravenous administration of MSCs induced a shift of monocyte phenotype in whole blood and enhanced the recruitment of granulocytic myeloid-derived cells, B cells (ASB), and T cells (CD8^+^) from circulation to the site of infection while reducing immune cell infiltration into bronchoalveolar space caused by H1N1 IAV infection. Despite this clear evidence of immunomodulatory activities during IAV infection, there was no reduction in mortality, possibly due to infection of MSCs by H1N1 IAV, limiting their overall therapeutic potential.

By deep profiling the immune landscape in whole blood and BAL of IAV-infected mice, we showed evidence that MSC treatment resulted in increases in recruitment of antibody-secreting B cells and CD8^+^ cytotoxic T cells into the lung. Early activation of B cells contributes to a better host response against influenza viral infection by rapidly generating neutralizing antibodies to blunt the initial infection and help in inflammation resolution (Lam and Baumgarth, 2019). Cytotoxic T-cell immunity is also important in limiting the severity and transmission of influenza infection by specifically eliminating infected cells (La Gruta and Turner, 2014). Although we observed a significant impact of MSCs on the host immune response, there was no reduction in virus titer in BALF and mortality in IAV-infected mice treated with MSCs. Nevertheless, as none of the previous studies conducted comprehensive immune profiling of IAV-infected animals after MSC treatment, our study provided clear and definitive evidence of early immunomodulatory bioactivities of MSCs in an IAV-induced lung injury model.

Chan et al. (2016) reported that MSCs, as an adjunctive therapy with an antiviral agent, significantly reduced the impairment of alveolar fluid clearance induced by avian IAV (H5N1) infection and prevented or reduced H5N1-associated acute lung injury. In addition, MSCs were shown to inhibit the replication of H9N2 IAV *in vitro* by reducing the expression of viral receptors on host cells and enhancing the antiviral activity of interferons (Li et al., 2016). In contrast, Darwish and colleagues demonstrated that the administration of murine or human MSCs, either prophylactically or therapeutically, failed to improve survival, decrease pulmonary inflammation, or prevent ALI in H1N1-infected mice (Darwish et al., 2013). Another recent study further confirmed that mouse or human MSC treatment did not significantly affect H1N1 viral proliferation or mouse mortality in H1N1-induced ALI, despite the evidence of biological activity by blocking influenza-induced thrombocytosis (Gotts et al., 2014).

In virus-induced ALI, viral tropism needs to be carefully considered for the evaluation of MSCs efficacy. We reported in this study that nearly all MSCs (99.43% ± 0.21%) exhibited high expression of α-2,6-linked SA (measured by SNA, Figure 7A), H1N1 IAV binding receptors and were highly permissive to infection by H1N1 (swine-origin) strain. The upregulated interferon-stimulated genes such as Mx1, IFIT1, and IFITM3 in MSCs, after H1N1 IAV infection, failed to provide sufficient protection for MSCs from the H1N1 virus-induced cytopathic effects and subsequently cell death. Therefore, we observed a similar lack of MSC efficacy in reducing H1N1 IAV-induced mortality in our study, consistent with other studies reported to date (Darwish et al., 2013; Gotts et al., 2014). In contrast, the encouraging results of MSCs in avian IAV (H5/H9 strains) (Chan et al., 2016; Li et al., 2016) induced ALI and its associated mortality could be due to the different strains used, as a significantly lower expression of avian IAV receptor (2,3-linked SA, measured by MAA, Figure 7A) is found on MSCs. It is likely that MSCs could be primed by avian IAV but not fully susceptible to the cytopathic effects or significant cell death, which could explain the better beneficial effect seen in avian IAV (H5 or H9 strain)-induced models. The lower susceptibility of MSCs to avian IAV infection may mirror the effect of viral mimic priming we previously reported in other viral diseases (Souza-Moreira et al., 2022). All in all, the function and efficacy of MSCs may vary in ALI induced by different strains of respiratory viruses, and a more thorough understanding of the effect of MSCs on immune responses and viral susceptibility is needed to improve MSC-based therapy.

## 5 Conclusion

In conclusion, we have provided thorough *in vivo* insights that MSCs were capable of inducing significant immune modulatory effects on phenotypic shifts and mobilization of granulocytes, B-cell, and T-cell subsets, evolving the immune response elicited by viral infection. When employing MSCs as a therapy in the context of H1N1 infection, IAV strain-specific tropism needs to be carefully considered.

## Data Availability

The raw data supporting the conclusion of this article will be made available by the authors, without undue reservation.
