# Supplementary material for: Mesenchymal stem cells induce dynamic immunomodulation of airway and systemic immune cells in vivo but do not improve survival for mice with H1N1 virus-induced acute lung injury
Source: Front Bioeng Biotechnol. 2023 Jun 8;11:1203387. doi: 10.3389/fbioe.2023.1203387 (PMC10285296; doi:10.3389/fbioe.2023.1203387)
Supplement: Supplementary file 1 [file DataSheet1.docx]

Supplementary Material

**Mesenchymal Stem Cells Induces Dynamic Immunomodulation of Airway and Systemic Immune Cells in mice with H1N1 Virus-Induced Acute Lung Injury**

**Yuan Tan1, 2†, Yan Wang1†; Luciana Souza-Moreira1; Chi Wang1; Aidan B.P. Murray1; Mahmoud Salkhordeh1; Maria Florian1; Lauralyn McIntyre1,3 Duncan J. Stewart1, 2 and Shirley H.J. Mei1***

*** Correspondence:**

Shirley H.J. Mei
smei@ohri.ca

# Supplementary Figures and Tables

## Supplementary Figures


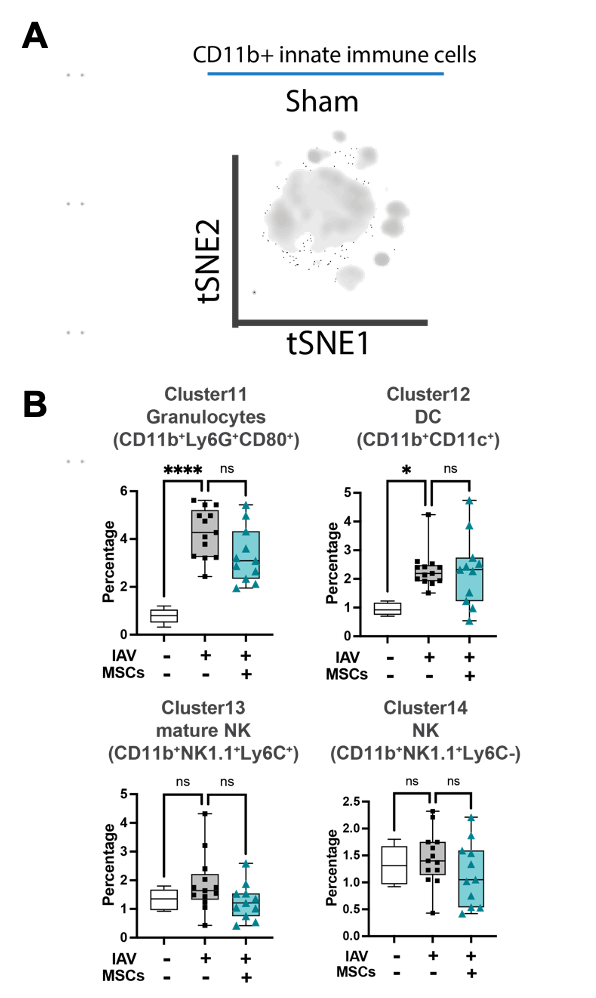


**Supplementary Figure 1. Mass Cytometry Analysis of circulating innate immune cells in H1N1-induced ALI. Related to Figure 1**

(A). t-SNE plot of sham group.

(B). Quantification of cell subpopulation.

n = 6 of sham, n=13 of IAV/vehicle- and n=11 of IAV/MSC-treated animals with the data shown as Dunnett's box-and-whisker plots. ∗p < 0.05, ∗∗p < 0.01, ∗∗∗p < 0.005, ∗∗∗∗p < 0.001.

**
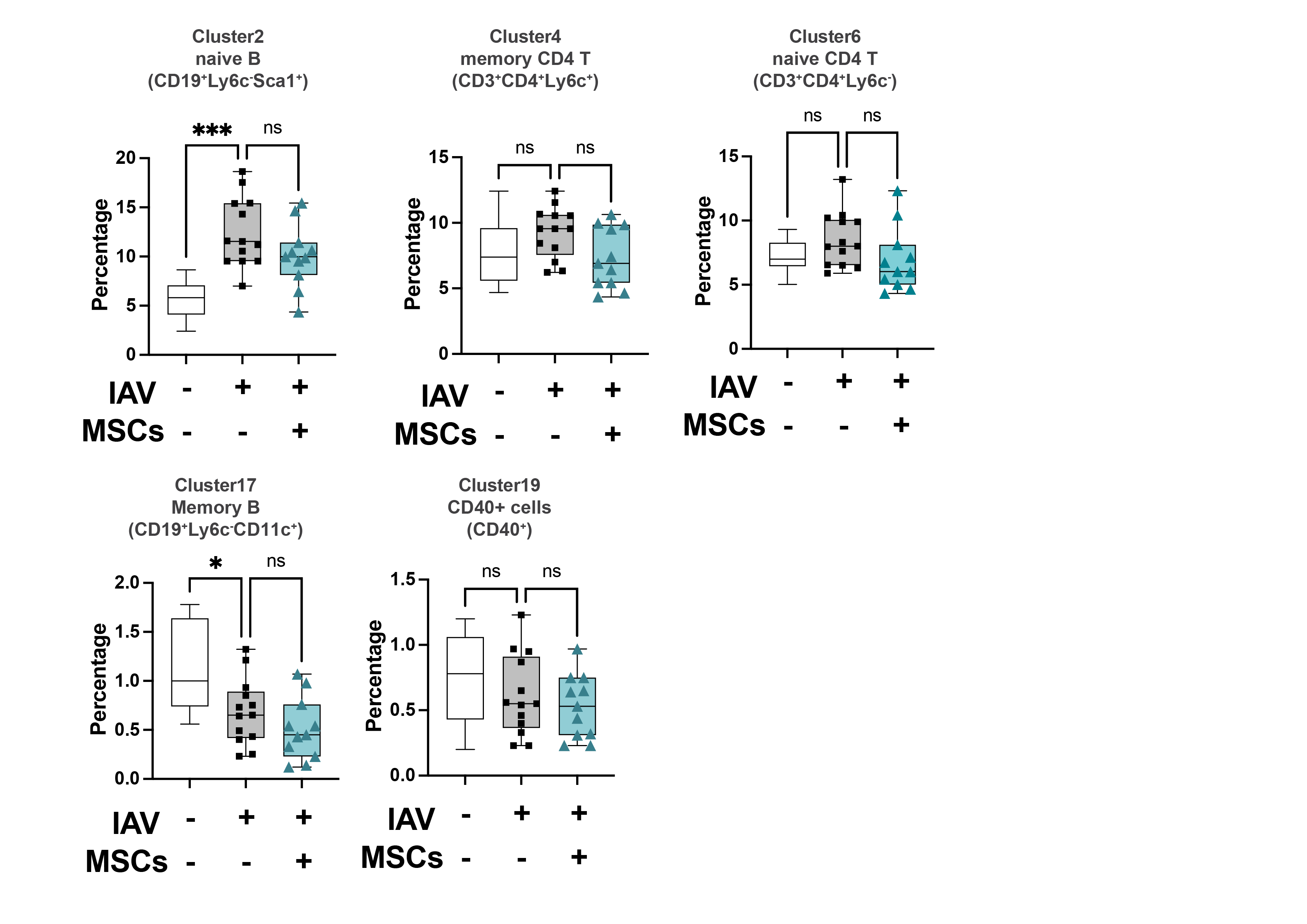
**

**Supplementary Figure 2.** **Mass Cytometry Analysis of circulating adaptive immune cell in H1N1-induced ALI. Related to Figure 2**

Quantification of cell subpopulation.

n = 6 of sham, n=13 of IAV/vehicle- and n=11 of IAV/MSC-treated animals with the data shown as Dunnett's box-and-whisker plots. ∗p<0.05, ∗∗p < 0.01, ∗∗∗p < 0.005, ∗∗∗∗p <0.001.

.

**
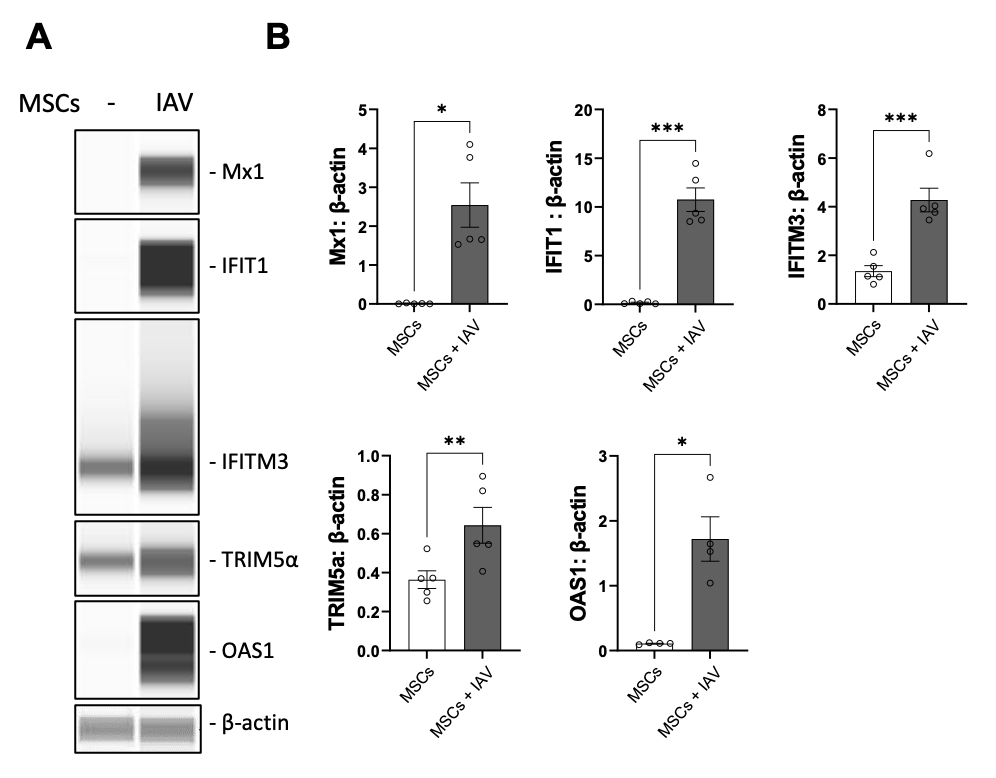
**

**Supplementary Figure 3.** **Upregulation of antiviral protein expression in MSCs when infected by H1N1 IAV.**

(A) Western blot analysis showed increased antiviral protein expressions in MSCs after in vitro exposure to H1N1 (MOI 0.1, 24h exposure ).

(B) Densitometry analysis of protein expression detected using automated western blot. n = 5 with the data shown as mean ± SEM, two-tailed t-test ∗p <0.05, ∗∗p <0.01, *** p <0.005.

.

## Table

**Table 1. List of antibodies.**

| ANTIBODY - HUMAN | Supplier | Catalogue # | RRID |
| --- | --- | --- | --- |
| RNase L (D4B4J) Rabbit mAb | Cell Signaling Technology | 27281 | AB_2798941 |
| OAS1 (D1W3A)  Rabbit mAb | Cell Signaling Technology | 14498 | AB_2798498 |
| MX1 (D3W7I)  Rabbit mAb | Cell Signaling Technology | 37849 | AB_2799122 |
| IFITM3 (D8E8G) XP® Rabbit mAb | Cell Signaling Technology | 59212 | AB_2799561 |
| TRIM5α (D6Z8L) Rabbit mAb | Cell Signaling Technology | 14326 | AB_2798451 |
| IFIT1 (D2X9Z)  Rabbit mAb | Cell Signaling Technology | 14769 | AB_2783869 |
| Monoclonal Anti-β-Actin, mouse, clone AC-15, ascites fluid | Sigma-Aldrich | A5441 | AB_476744 |
|  | | | |
| ANTIBODY - MOUSE | Supplier | Catalogue # | RRID |
| Anti-Mouse CD3e  (145-2C11)-152Sm | Fluidigm | 3152004C | N/A |
| Anti-Mouse CD4  (RM4-5)-145Nd | Fluidigm | 3145002C | N/A |
| Anti-Mouse CD8a  (53-6.7)-168Er | Fluidigm | 3168003C | N/A |
| Anti-Mouse CD279  (RMP1-30)-159Tb | Fluidigm | 3159006C | N/A |
| Anti-Mouse CD11b  (M1/70)-148Nd | Fluidigm | 3148003C | N/A |
| Anti-Mouse CD11c  (N418)-209Bi | Fluidigm | 3209005C | N/A |
| Anti-Mouse I-A/IE  (M5/114.15.2)-  174Yb | Fluidigm | 3174003C | N/A |
| Anti-Mouse/Rat  CD40 (HM40-3)-  161Dy | Fluidigm | 3161020C | N/A |
| Anti-Mouse CD80  (16-10A1)-171Yb | Fluidigm | 3171008C | N/A |
| Anti-Mouse CD86  (GL1)-172Yb | Fluidigm | 3172016C | N/A |
| Anti-Mouse Ly-6C  (HK1.4)-162D | Fluidigm | 3162014C | N/A |
| Anti-Mouse Ly-6G  (1A8)-141Pr | Fluidigm | 3141008C | N/A |
| Anti-Mouse CD206/  MMR (C068C2)-  169Tm | Fluidigm | 3169021C | N/A |
| Anti-Mouse CD64  (X54-5/7.1)-151Eu | Fluidigm | 3151012C | N/A |
| Anti-Human/Mouse  CD27 (LG.3A10)-150Nd | Fluidigm | 3150017C | N/A |
| Anti-Mouse Ly-6A/E  (Sca-1) (D7)-164Dy | Fluidigm | 3164005C | N/A |
| Anti-Mouse CD45  (30-F11)-89Y | Fluidigm | 3089005C | N/A |
| Anti-Mouse CD274/  PD-L1 (10F.9G2)-153Eu | Fluidigm | 3153016C | N/A |
| Anti-Mouse F4/80  (BM8)-146Nd | Fluidigm | 3146008C | N/A |
| Anti-Mouse NK1.1  (PK136)-170Er | Fluidigm | 3170002C | N/A |
| Anti-Mouse CD19  (6D5)-166Er | Fluidigm | 3166015C | N/A |
| Anti-Mouse CD69  (H1.2F3)-143Nd | Fluidigm | 3143004C | N/A |
| Anti-Mouse CD45  (30-F11)-89Y | Fluidigm | 3089005C | N/A |
